# Analysis of risk factors for recurrence after middle meningeal artery embolization combined with burr hole drainage in patients with chronic subdural hematoma

**DOI:** 10.1097/MD.0000000000045116

**Published:** 2025-10-24

**Authors:** Wen Cheng, Quanlong Yang, Xiaodong Yuan, Jiangbin Wu

**Affiliations:** a Neurovascular Interventional Therapy Department, Affiliated Hospital and Clinical Medical College of Chengdu University, Chengdu, Sichuan, China.

**Keywords:** chronic subdural hematoma, hyperbaric oxygen therapy, middle meningeal artery embolization, predictive model, recurrence

## Abstract

Chronic subdural hematoma (CSDH) is a common disease in neurosurgery. Middle meningeal artery embolization (MMAE) combined with subdural drainage has become the mainstream treatment option, significantly reducing surgical trauma and hospitalization time. However, in clinical practice, a certain proportion of patients still experience postoperative recurrence, which not only affects patient prognosis but also increases the burden on medical care. Currently, the risk factors for recurrence after this combined surgical procedure have not been fully identified, and there is an urgent need for in-depth research to provide scientific evidence for clinical intervention. To investigate the risk factors for recurrence in patients with CSDH after MMAE combined with subdural drainage and to establish a predictive model. A total of 211 patients were included in this study, among whom 35 patients experienced postoperative hematoma recurrence. The patients enrolled in this study were randomly divided into a training set and a validation set in a 7:3 ratio, with 147 patients in the training set and 64 patients in the validation set. This study collected patients’ medical histories, onset conditions, and relevant information during hospitalization to study the factors affecting recurrence in patients with CSDH after MMAE combined with subdural drainage, and established a predictive model. Potentially relevant factors were included in univariate logistic regression analysis, and the results showed that gender, postoperative drainage volume, postoperative statin use, postoperative hyperbaric oxygen therapy, admission Glasgow Coma Scale score, and preoperative hematoma volume were potential risk factors for recurrence in patients with CSDH who underwent MMAE combined with subdural burr hole drainage, *P* < .2. Further inclusion of the obtained data in a multivariate retrospective analysis revealed that postoperative drainage volume, postoperative hyperbaric oxygen therapy, admission Glasgow Coma Scale score, and preoperative hematoma volume were independent risk factors for recurrence in patients with CSDH who underwent MMAE combined with subdural burr hole drainage (*P* < .05). The predictive model developed in this study can help neurosurgeons accurately identify high-risk CSDH patients who are likely to experience recurrence after MMAE combined with subdural burr hole drainage.

## 1. Introduction

Chronic subdural hematoma (CSDH) is a hidden neurosurgical disease characterized by chronic accumulation of blood between the dura mater and arachnoid membrane. It is common in elderly people, those who take anticoagulants long-term, and patients with sequelae of traumatic brain injury.^[1–4]^ Epidemiological studies show that with the aging of the global population, the incidence of CSDH is on the rise, particularly among the elderly. Long-term use of anticoagulants or antiplatelet drugs interferes with the body’s normal clotting mechanism and increases the risk of subdural hemorrhage.^[5,6]^ With the acceleration of global aging and the widespread use of antiplatelet and anticoagulant therapies, the incidence of CSDH has been increasing year by year, becoming an important disease that threatens the neurological function and quality of life of middle-aged and elderly patients.^[7]^ In addition, a history of minor head trauma is also an important contributing factor to CSDH. Even seemingly minor head impacts can cause rupture of the pontine vein, which can gradually develop into a chronic hematoma. Currently, middle meningeal artery embolization (MMAE) combined with subdural drainage has become the first-line clinical treatment option due to its minimally invasive advantages, which significantly reduce the risk of complications associated with traditional craniotomy by blocking the blood supply to the hematoma and removing the hematoma.^[8–10]^ Although this combination therapy effectively improved patient prognosis, postoperative recurrence remains a key issue that needs to be addressed. Research by Hirohisa et al indicates that treatment with MMAE alone for CSDH is safe and feasible, as patients did not experience recurrence or complications postoperatively. However, it is worth noting that their study sample size was small and may not be representative of all patients.^[11]^ Lampros et al found that 9% of patients experienced recurrence following single-port or double-drill drainage procedures.^[12]^ In the study by Jeon et al, 10.17% of cerebral hemispheres experienced recurrence within 6 months following burr hole drainage.^[13]^ Clinical data show that the recurrence rate of CSDH after MMAE combined with burr hole drainage fluctuates between 5% and 20%. Recurrence not only requires patients to undergo secondary or even multiple surgeries, prolonging their hospital stay, but may also lead to serious sequelae such as chronic brain damage and cognitive impairment.^[14–17]^ Previous studies have mostly focused on risk factors for recurrence associated with a single treatment modality, such as advanced age, coagulation abnormalities, and the extent of brain atrophy. However, there are still significant gaps in research on the recurrence mechanism of this combined therapy, namely middle meningeal artery (MMA) combined drilling and drainage. There is currently no consensus in the literature on the pathophysiological mechanisms of rebleeding from hematomas. Some studies suggest that impaired vascular endothelial repair is a key factor, while other scholars emphasize the role of local microenvironmental imbalance in the hematoma cavity.

In addition, existing studies have limitations in the screening and validation of risk factors. Most retrospective analyses have small sample sizes and lack support from multicenter, large-sample data. Furthermore, the variables included in different studies varied significantly, resulting in insufficient comparability of results. For example, there is no consensus on the association between preoperative imaging characteristics (such as hematoma compartmentalization and density heterogeneity) and recurrence, or on the impact of anticoagulant management strategies on prognosis.^[18,19]^ This research status severely limits the formulation of clinical precision treatment strategies. There is an urgent need for systematic analysis to identify key risk factors and provide evidence-based medical evidence for optimizing perioperative management and reducing the risk of recurrence. This study addresses the aforementioned clinical and academic challenges by conducting a retrospective cohort analysis to systematically investigate the potential risk factors for CSDH recurrence following MMAE combined with subdural drainage. The aim is to fill existing research gaps and provide more targeted risk assessment tools and intervention strategies for clinical decision-making.

## 2. Materials and methods

### 2.1. Research subjects

The study encompassed a total of 211 patients diagnosed with CSDH. These patients were admitted to the Neurovascular Interventional Therapy Department, Affiliated Hospital and Clinical Medical College of Chengdu University, located in Chengdu, Sichuan, China, for surgical treatment during the period from June 2022 to June 2024. Among them, there were 118 male and 93 female patients.

### 2.2. Research methods

This study used a retrospective case-control study design and collected data from a total of 211 patients. Among them, 35 patients experienced recurrence after undergoing MMAE combined with subdural drainage. Collect patients’ medical history, smoking and drinking habits, and relevant information during their hospital stay to study the factors that affect the recurrence of postoperative hematoma.

### 2.3. Collect indicators

This study collected data on patients’ gender, age, hypertension, diabetes, coronary heart disease, smoking history, drinking history, history of head trauma, duration of illness, Glasgow Coma Scale (GCS) score at admission, preoperative hematoma volume, preoperative midline shift, preoperative platelet, postoperative drainage volume, postoperative statin use, and postoperative hyperbaric oxygen therapy. The raw data supporting the conclusions of this article will be made available by the authors, without undue reservation. The raw data has been uploaded to the attachments, see Raw Data S1, Supplemental Digital Content, https://links.lww.com/MD/Q300 for details.

### 2.4. Inclusion and exclusion criteria

#### 2.4.1. Inclusion criteria

Imaging examination, combined with medical history, symptoms, signs, and auxiliary examinations, is consistent with the diagnosis of CSDH; age ≥ 18 years old; all enrolled patients underwent MMAE combined with subdural drainage; clinical data is complete and intact; and Patients have no history of neurological diseases.

#### 2.4.2. Exclusion criteria

The patient has a history of head surgery; the patient has an autoimmune disease or malignant tumor; the patient has severe cardiac, hepatic, or renal dysfunction or mental disorders; the patient has coagulation disorders; and the patient is currently undergoing other treatments or participating in other clinical studies.

### 2.5. The relevant issues mentioned in this study

Surgical procedure: all patients enrolled in this study received MMAE combined with subdural drainage.

MMAE: first, local anesthesia is administered (intravenous anesthesia or endotracheal intubation under general anesthesia is performed when the patient is unable to cooperate). The patient lies supine on the angiography table. The puncture site is routinely disinfected and draped. The Seldinger technique is used for puncture, and an 8F sheath is placed. Whole-body heparinisation (40 mg bolus injection) is performed. A 0.035-inch guidewire + 5F 125 cm angiography guidewire is used to guide the guidewire to the external carotid artery under ultrasound guidance. Using a microcatheter + Marathon microcatheter, the intermediate catheter was selectively advanced to the maxillary internal artery and a roadmap was established; using a microcatheter, the Marathon, Apollo microcatheters to the frontal, parietal, and occipital branches of the middle meningeal artery, respectively. After confirming with microcatheter push-through angiography, Onyx glue was slowly injected into the branches in sequence, totaling approximately 1.8 mL. After satisfactory angiographic embolization, the puncture site was closed with a vascular sealer to stop bleeding.

Burr hole drainage: after local anesthesia (intravenous anesthesia or endotracheal intubation general anesthesia cannot be performed when the patient is unable to cooperate), the patient is placed in a supine position. Using cranial CT imaging for localization, the puncture and catheter insertion point is determined at the intersection of the posterior one-third of the maximum plane of the hematoma and the scalp. During puncture, important blood vessels and functional areas are avoided. Perform routine disinfection and drape the area; administer local infiltration anesthesia; make an incision approximately 2 to 3 cm long at the puncture site, cut through to the skull, and peel back the periosteum. Use a drilling device (diameter 1–1.5 cm) to drill through the skull, then electrocoagulate the dura mater to stop bleeding. Make a cross-shaped incision in the dura mater and insert a size 10 silicone tube into the subdural space. If bloody drainage fluid is observed, continue to slowly advance the tube toward the forehead until the silicone tube reaches the predetermined insertion depth. Irrigate the hematoma cavity with physiological saline using a pulsed irrigation technique. Once the irrigation fluid becomes clear, remove the drainage tube. Use the same method to irrigate the temporal, parietal, and occipital regions. Finally, insert the drain to the predetermined depth in the forehead and confirm that drainage is unobstructed. Close the incision layer by layer and secure the drain to the drainage device.

Postoperative hyperbaric oxygen therapy: hyperbaric oxygen therapy was initiated 2 days postoperatively using a pure oxygen chamber. Set the treatment pressure, decompression time, and stabilization time to 0.20 MPa, 20 minutes, and 20 minutes, respectively, once per day.

### 2.6. Model construction and validation

To enable the model to learn underlying patterns, relationships, or characteristics from the data, this study created a training set. To rigorously evaluate the model’s performance on unseen data and prevent situations where the model performs well only on training data but poorly on new data, a validation set was established in this study. Patients were randomly divided into a training set and a validation set at a ratio of 7:3. Based on the training set data, we 1st performed univariate logistic regression analysis on the collected data to determine the potential risk factors for recurrence in patients with CSDH who underwent MMAE combined with burr hole drainage. In the univariate logistic regression analysis, exposure factors with *P* ≤ .2 were selected and included in the multi-factor analysis^[20,21]^; Subsequently, multivariate analysis was conducted to identify independent risk factors for recurrence in patients with CSDH who underwent MMAE combined with burr hole drainage, with *P* < .05 indicating a statistically significant difference. Finally, a risk prediction nomogram model was constructed for CSDH patients undergoing MMAE combined with subdural burr hole drainage, based on the identified independent predictors. The model was then validated internally to further confirm the reliability of the predictive model developed in this study. Internal validation of the training set was performed using the Bootstrap method in R software (with 1000 repeated samples). The calibration curve was employed to assess the agreement between the model’s predicted recurrence probability and the actual incidence rate. The validation set was not involved in model training at any stage and is used solely for external validation. Evaluate the model’s discriminatory power by calculating the area under the curve (AUC) value of the validation set. AUC is used to evaluate model performance. Calibration of the training set for the measurement model using the calibration curve. The model’s training set was also subjected to decision curves analysis (DCA) by calculating the net gains within the threshold probability range.

### 2.7. Statistical methods

This study used SPSS 25 software (IBM Corporation, Armonk) and R software (version 4.3.3; http://www.Rproject.org) to process and statistically analyze the data. Quantitative information that conformed to normal distribution was expressed as mean ± standard deviation, and differences between groups were analyzed using the independent samples *t*-test. Comparisons between groups that do not obey a normal distribution are made using nonparametric tests. Data for qualitative information were expressed as number of cases and percentages, and the chi-square test was used to determine if there were differences between groups. Predictive performance was evaluated with the AUC of the receiver operator characteristic (ROC). Multivariate binary logistic regression, nomograms, and calibration plots were executed with the “rms” package. Internal validation was performed using the “rms” package. DCA was performed using the “rmda” package.

## 3. Results

### 3.1. Table of values for relevant indicators in this study

This study assigned values to the variables. See Table 1 for details.

**Table 1:** Assignment table.

Name	Variable assignment and description
Group	Non-recurrence group: 0, Recurrence group: 1
Gender	Female: 0, Male: 1
Age	<65 yr: 0, ≥65 yr: 1
History of head injury	No: 0, Yes: 1
Course of illness	<3 months: 0, ≥3 months: 1
Preoperative midline deviation	<10 mm: 0, ≥10 mm: 1
Postoperative drainage volume	<200 mL: 0, ≥200 mL: 1

### 3.2. Comparison of baseline features between training set and validation set

This study randomly divide the patients included in this experimental study into a training set and a validation set at a ratio of 7:3. The training set consisted of 147 people, and the validation set consisted of 64 people. To facilitate a visual comparison of data characteristics between the training and validation sets, the key metrics are presented below: postoperative recurrence: training set – 23 individuals (15.65%); validation set – 12 individuals (18.75%). GCS score at admission: training set mean = 13.27 (SD = 1.53); validation set mean = 13.16 (SD = 1.60). Preoperative hematoma volume: training set mean = 91.45 mL (SD = 14.13); validation set mean = 89.80 mL (SD = 15.15). Preoperative platelet: training set mean = 213.34 × 10⁹/L (SD = 19.44); validation set mean = 211.72 × 10⁹/L (SD = 20.82). Gender (Male): training set – 83 (56.46%); validation set – 35 (54.69%). Age (≥65 years): training set – 98 (66.67%); validation set – 45 (70.31%). Hypertension: training set – 38 (25.85%); validation set – 17 (26.56%). Diabetes: training set – 23 (15.65%); validation set – 12 (18.75%). Coronary heart disease: training set – 27 (18.37%); validation set – 12 (18.75%). Smoking history: training set – 37 (25.17%); validation set – 17 (26.56%). Drinking history: training set – 77 (52.38%); validation set – 37 (26.56%). History of head trauma: training set - 96 (65.31%); validation set – 41 (64.06%). Duration of illness (≥3 months): training set – 98 (66.67%); validation set – 41 (64.06%). Postoperative drainage volume (≥200 mL): training set – 44 (29.93%); validation set – 25 (39.06%). Postoperative statin use: training set – 89 patients (60.54%); validation set – 32 patients (50.00%). Postoperative hyperbaric oxygen therapy: training set – 88 patients (59.86%); validation set – 35 patients (54.69%).

The general data of the 2 groups of patients were included in the statistical analysis: *P* > .05, indicating that there were no statistically significant differences in baseline characteristics between the 2 groups. See Table 2 for details.

**Table 2:** Comparison of baseline features between training set and validation set.

Variables	Total (n = 211)	Validation set (n = 64)	Training set (n = 147)	Statistic	*P*
GCS score upon admission	13.23 ± 1.55	13.16 ± 1.60	13.27 ± 1.53	*t* = −0.47	.639
Preoperative hematoma volume	90.95 ± 14.43	89.80 ± 15.15	91.45 ± 14.13	*t* = −0.76	.446
Preoperative PLT	212.85 ± 19.83	211.72 ± 20.82	213.34 ± 19.44	*t* = −0.55	.586
Group, n (%)				χ² = 0.31	.577
0	176 (83.41)	52 (81.25)	124 (84.35)		
1	35 (16.59)	12 (18.75)	23 (15.65)		
Gender, n (%)				χ² = 0.06	.811
0	93 (44.08)	29 (45.31)	64 (43.54)		
1	118 (55.92)	35 (54.69)	83 (56.46)		
Age, n (%)				χ² = 0.27	.602
0	68 (32.23)	19 (29.69)	49 (33.33)		
1	143 (67.77)	45 (70.31)	98 (66.67)		
Hypertension, n (%)				χ² = 0.01	.914
0	156 (73.93)	47 (73.44)	109 (74.15)		
1	55 (26.07)	17 (26.56)	38 (25.85)		
Diabetes mellitus, n (%)				χ² = 0.31	.577
0	176 (83.41)	52 (81.25)	124 (84.35)		
1	35 (16.59)	12 (18.75)	23 (15.65)		
Coronary heart disease, n (%)				χ² = 0.00	.948
0	172 (81.52)	52 (81.25)	120 (81.63)		
1	39 (18.48)	12 (18.75)	27 (18.37)		
Smoking history, n (%)				χ² = 0.05	.831
0	157 (74.41)	47 (73.44)	110 (74.83)		
1	54 (25.59)	17 (26.56)	37 (25.17)		
Drinking history, n (%)				χ² = 0.53	.467
0	97 (45.97)	27 (42.19)	70 (47.62)		
1	114 (54.03)	37 (57.81)	77 (52.38)		
History of head injury, n (%)				χ² = 0.03	.862
0	74 (35.07)	23 (35.94)	51 (34.69)		
1	137 (64.93)	41 (64.06)	96 (65.31)		
Course of illness, n (%)				χ² = 0.13	.714
0	72 (34.12)	23 (35.94)	49 (33.33)		
1	139 (65.88)	41 (64.06)	98 (66.67)		
Postoperative drainage volume, n (%)				χ² = 1.69	.194
0	142 (67.30)	39 (60.94)	103 (70.07)		
1	69 (32.70)	25 (39.06)	44 (29.93)		
Postoperative statin therapy n (%)				χ² = 2.03	.155
0	90 (42.65)	32 (50.00)	58 (39.46)		
1	121 (57.35)	32 (50.00)	89 (60.54)		
Postoperative hyperbaric oxygen therapy, n (%)				χ² = 0.49	.483
0	88 (41.71)	29 (45.31)	59 (40.14)		
1	123 (58.29)	35 (54.69)	88 (59.86)		

GCS = Glasgow Coma Scale, PLT = platelet.

### 3.3. Single factor analysis

A total of 147 patients in the training set were included in the statistical analysis, among whom 23 patients experienced postoperative hematoma recurrence. Potentially relevant factors were included in univariate logistic regression: gender, postoperative drainage volume, postoperative statin use, postoperative hyperbaric oxygen therapy, admission GCS score, and preoperative hematoma volume were potential risk factors for recurrence in patients with CSDH who underwent MMAE combined with burr hole drainage, *P* ≤ .2. See Table 3 for details.

**Table 3:** Single-factor logistic regression analysis based on the training set.

Variables	β	SE	*Z*	*P*	OR (95% CI)
Gender					
0					1.00 (Reference)
1	1.18	0.54	2.21	.027	3.27 (1.14–9.35)
Age					
0					1.00 (Reference)
1	−0.30	0.47	−0.64	.522	0.74 (0.30–1.86)
Hypertension					
0					1.00 (Reference)
1	0.27	0.50	0.55	.585	1.31 (0.49–3.49)
Diabetes mellitus					
0					1.00 (Reference)
1	0.15	0.60	0.25	.802	1.16 (0.36–3.80)
Coronary heart disease					
0					1.00 (Reference)
1	−0.98	0.77	−1.26	.207	0.38 (0.08–1.72)
Smoking history					
0					1.00 (Reference)
1	0.06	0.52	0.11	.912	1.06 (0.38–2.92)
Drinking history					
0					1.00 (Reference)
1	−0.01	0.45	−0.02	.983	0.99 (0.41–2.41)
History of head injury					
0					1.00 (Reference)
1	−0.00	0.48	−0.01	.992	1.00 (0.39–2.53)
Course of illness					
0					1.00 (Reference)
1	0.41	0.51	0.80	.424	1.50 (0.55–4.09)
Postoperative drainage volume					
0					1.00 (Reference)
1	1.58	0.48	3.33	<.001	4.87 (1.92–12.39)
Postoperative statin therapy					
0					1.00 (Reference)
1	−1.26	0.48	−2.65	.008	0.28 (0.11–0.72)
Postoperative hyperbaric oxygen therapy					
0					1.00 (Reference)
1	−1.00	0.47	−2.15	.031	0.37 (0.15–0.91)
GCS score upon admission	−1.07	0.25	−4.33	<.001	0.34 (0.21–0.56)
Preoperative hematoma volume	0.06	0.02	3.49	<.001	1.06 (1.03–1.09)
Preoperative PLT	0.01	0.01	0.68	.496	1.01 (0.99–1.03)

95% CI = 95% confidence interval, GCS = Glasgow Coma Scale, OR = odds ratio, PLT = platelet.

### 3.4. Multifactorial analysis

The risk factors identified in the univariate analysis were further incorporated into a multivariate analysis, which showed that postoperative drainage volume, postoperative hyperbaric oxygen therapy, GCS score at admission, and preoperative hematoma volume were independent risk factors for recurrence in patients with CSDH who underwent MMAE combined with burr hole drainage. See Table 4 for details.

**Table 4:** Multi-factor logistic regression analysis based on the training set.

Variables	β	SE	*Z*	*P*	OR (95% CI)
Postoperative drainage volume					
0					1.00 (Reference)
1	1.39	0.64	2.18	.029	4.03 (1.15–14.09)
Postoperative hyperbaric oxygen therapy					
0					1.00 (Reference)
1	−1.48	0.64	−2.30	.022	0.23 (0.06–0.80)
GCS score upon admission	−0.98	0.27	−3.60	<.001	0.37 (0.22–0.64)
Preoperative hematoma volume	0.06	0.02	2.78	.005	1.06 (1.02–1.11)

95% CI = 95% confidence interval, GCS = Glasgow Coma Scale, OR = odds ratio.

### 3.5. Drawing a nomogram

**Figure 1.**
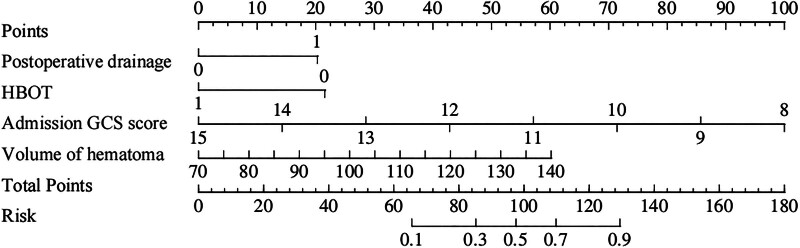
Nomogram prediction of recurrence risk after MMAE combined with burr hole drainage in patients with CSDH. CSDH = chronic subdural hematoma, GCS = GCS = Glasgow Coma Scale, MMAE = middle meningeal artery embolization.

**Figure 2.**
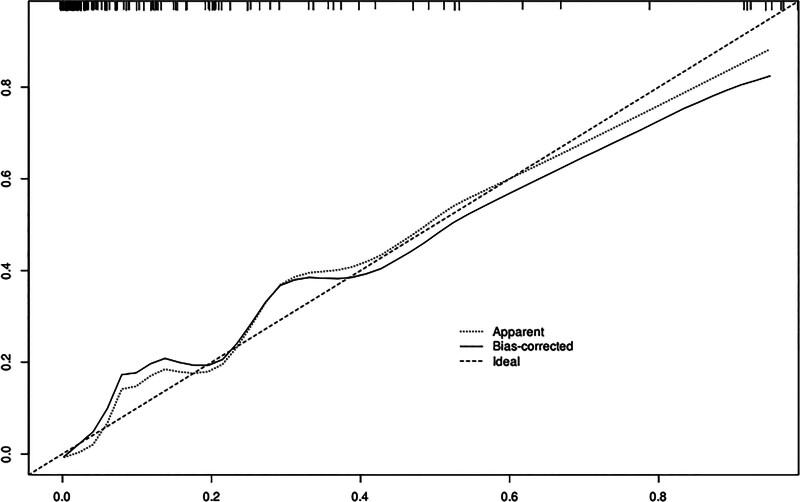
Internal validation of nomograms: calibration curves.

**Figure 3.**
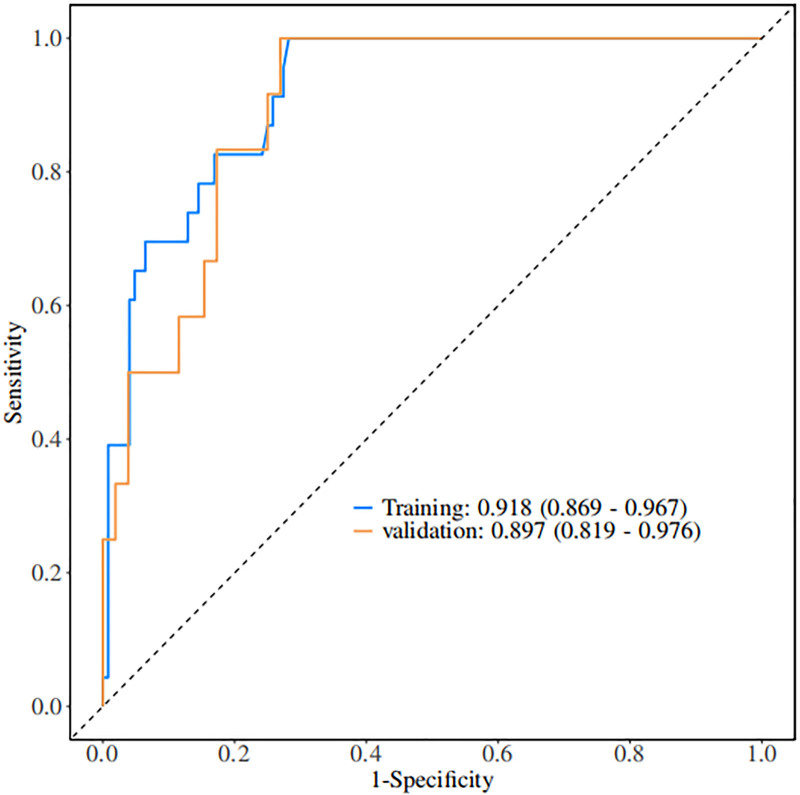
Nomogram validation: ROC. ROC = receiver operating characteristic.

**Figure 4.**
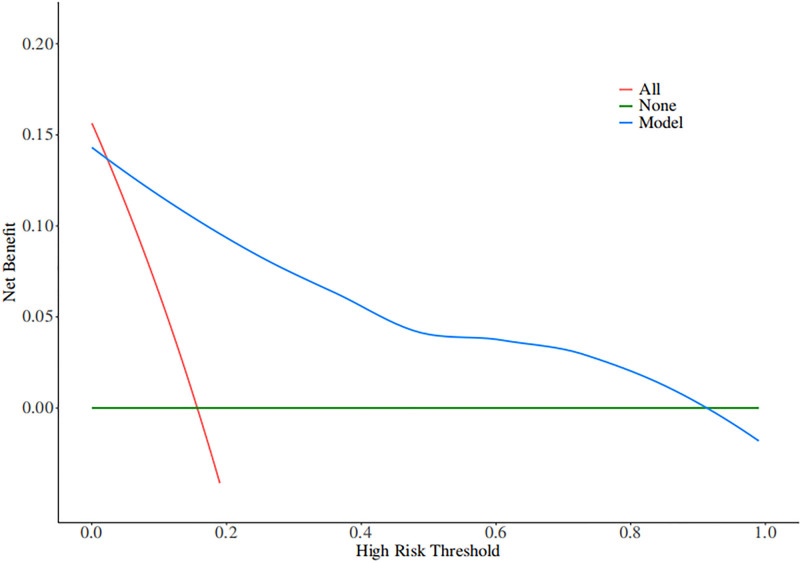
Decision curve in nomogram model.

Based on independent predictors tested by multivariate logistic regression analysis, a nomogram was constructed to predict the risk of recurrence in patients with CSDH after MMAE combined with burr hole drainage, see Figure 1. Assign a Nomo score to each independent risk factor. The total score is calculated based on the patient’s clinical characteristics and plotted on the total points axis. The value on the risk axis corresponding to the vertical downward direction is the probability of hematoma recurrence of the patient after operation. The scores for each independent predictor correspond to the maximum scores for each independent predictor. The total score for each subject is the sum of the scores for each independent predictor. The probability of recurrence in patients with CSDH who undergo MMAE combined with burr hole drainage is determined by the total score on the risk axis. The model was then validated internally using the Bootstrap method in R software to repeat sampling 1000 times to validate the nomogram. The calibration curve is close to the ideal curve, indicating that the nomogram predicts the incidence of recurrence in patients with CSDH after MMAE combined with burr hole drainage with a high degree of consistency with the actual incidence, demonstrating good predictive performance, see Figure 2. The ROC curve of the training set of the nomogram had an AUC of 0.918 (95% confidence interval = 0.869–0.967); the ROC curve of the validation set had an AUC of 0.897 (95% confidence interval = 0.819–0.976), see Figure 3. This nomogram has good discriminatory power for identifying patients with CSDH who are at high risk of recurrence after MMAE combined with burr hole drainage. The DCA of this nomogram shows that: In this nomogram, when an individual’s threshold probability is >.05, the model provides more net benefits than the “intervene in everyone” or “do not intervene in anyone” strategies. This conclusion shows that the nomogram model has good clinical application value in predicting recurrence in patients with CSDH after MMAE combined with subdural burr hole drainage, see Figure 4.

## 4. Discussion

Although MMAE combined with burr hole drainage provides a new minimally invasive treatment option for patients with CSDH, postoperative recurrence remains a key issue affecting long-term prognosis.^[1,2]^ Currently, there are still many controversies in the academic community regarding the mechanism of recurrence of this combined surgical procedure: on the one hand, the association between traditional risk factors such as advanced age and brain atrophy and recurrence has not yet been fully clarified. Some studies suggest that these factors may exacerbate hematoma cavity closure disorders, but the specific pathophysiological pathways remain unclear. On the other hand, after the introduction of new treatment technologies, there has been a lack of systematic discussion on the impact of surgical-related factors such as incomplete MMAE and drainage tube retention time on recurrence. This study, through multidimensional analysis, integrated clinical characteristics, imaging parameters, and perioperative management variables for the 1st time, revealing potential risk factors associated with recurrence. The following discussion will combine previous literature with data from this study to explore these risk factors in depth from the perspectives of pathological mechanisms, clinical interventions, and future research directions.

### 4.1. Exploring the mechanisms of independent risk factors for postoperative recurrence

This study revealed that postoperative drainage volume, postoperative hyperbaric oxygen therapy, admission GCS score, and preoperative hematoma volume were independent risk factors for recurrence after MMAE combined with burr hole drainage. These factors affect hematoma outcome through complex pathophysiological mechanisms.

Postoperative drainage volume directly reflects the closure status and pressure balance of the hematoma cavity. Excessive drainage may lead to intracranial negative pressure, inducing pontine vein rupture or rebleeding. Insufficient drainage means residual hematoma, local inflammatory mediators continue to stimulate the meninges, promote new blood vessel formation, and increase the risk of hematoma recurrence. The association between this pressure imbalance and rebleeding in the hematoma cavity has been partially verified in traditional burr hole drainage studies. However, under the MMA combined treatment model, the synergistic effect of vascular embolization on pressure regulation is not yet fully understood and warrants further exploration.^[18]^ Although postoperative hyperbaric oxygen therapy promotes hematoma absorption by improving cerebral oxygen metabolism, its double-edged sword effect should not be overlooked. From a molecular biology perspective, under high-pressure conditions with a sudden increase in oxygen partial pressure, the expression of hypoxia-inducible factor-1α in brain tissue is upregulated, thereby activating the downstream vascular endothelial growth factor (VEGF) transcription pathway, promoting the proliferation, migration, and lumen formation of vascular endothelial cells, and accelerating the repair of damaged blood vessels. However, abnormal angiogenesis leads to a lack of pericellular coverage of the new blood vessels and disruption of the basement membrane structure. The high permeability of these “immature” blood vessels allows plasma components and red blood cells to leak into the subdural space, forming the pathological basis for blood accumulation. Clinical studies have shown that VEGF levels may increase by 2 to 3 times after hyperbaric oxygen therapy compared to pretreatment levels, and there is a significant positive correlation with hematoma recurrence (*r* = 0.68, *P* < .01).^[22]^ In terms of inflammation regulation, hyperbaric oxygen therapy exhibits dose-dependent characteristics in its regulation of the immune system. Moderate hyperbaric oxygen therapy can reduce pro-inflammatory factors such as tumor necrosis factor-α and interleukin-6 expression, thereby alleviating meningeal inflammatory responses and reducing vascular permeability. However, excessive suppression of immune function weakens the phagocytic action of macrophages on fibrin and cell debris in hematomas, hindering the organization and absorption of hematomas. Animal experiments have shown that continuous hyperbaric oxygen therapy for more than 7 days reduces macrophage activity in hematomas by 32% compared to the control group and slows down fibrinolysis. How this complex biological effect influences the risk of recurrence in this combined surgical procedure requires further clarification through molecular mechanism research, with particular attention to the temporal and pressure parameters of hyperbaric oxygen therapy and their dynamic association with the VEGF-inflammatory factor axis.^[23,24]^ In addition, the GCS score upon admission reflects the patient’s baseline neurological function. Severe neurological damage is often accompanied by impaired autoregulation of cerebral blood flow, leading to abnormal perfusion in the hematoma area. When cerebral blood flow dynamics remain disrupted, it is difficult to establish a stable internal environment in the hematoma cavity, increasing the risk of recurrence. In addition, excessive hematoma volume prior to surgery not only compresses surrounding brain tissue but may also compromise the integrity of the local blood–brain barrier, leading to plasma protein leakage and inflammatory cell infiltration, thereby creating a pathological microenvironment that promotes hematoma recurrence. This mechanism is similar to the conclusions of previous studies on single drainage techniques, but whether MMA combination therapy has a different effect on large hematomas still needs to be verified by more clinical data.^[25–27]^

Compared with traditional single subdural burr hole drainage or MMAE, this combined procedure can significantly reduce the risk of rebleeding in the hematoma cavity by precisely embolizing the main blood supply arteries such as the middle meningeal artery. In theory, it can effectively weaken some of the core risk factors for hematoma recurrence, such as continuous blood supply and pressure imbalance in the hematoma cavity.^[27]^ However, the innovative optimization of this treatment strategy has also given rise to a series of urgent clinical issues that need to be addressed: postoperative drainage management requires a precise balance between maintaining low pressure in the hematoma cavity and avoiding low intracranial pressure syndrome. Improper drainage control may lead to residual hematoma or poor brain re-expansion. The timing of hyperbaric oxygen therapy is equally critical. Premature intervention may exacerbate vascular permeability and lead to rebleeding, while delayed intervention may miss the optimal window of opportunity for promoting brain function recovery. This shift from single therapy to combined treatment models places higher demands on the diagnostic and treatment thinking of clinicians. There is an urgent need to reexamine the mechanisms of recurrence and explore the interactions between various influencing factors, thereby providing a solid theoretical basis for the development of individualized, precise intervention programs and promoting the advancement of CSDH treatment to a higher level.^[25,27]^

### 4.2. Clinical value of nomogram models

The nomogram quantifies independent risk factors such as postoperative drainage volume, hyperbaric oxygen therapy, GCS score at admission, and preoperative hematoma volume, transforming complex multivariate analysis into an intuitive and practical predictive tool. The core logic is to assign corresponding weights to each risk factor based on the regression coefficients of the logistic regression model and map them to the scoring axis of the nomogram. Clinicians simply need to locate the scores corresponding to each factor on the respective axes based on the patient’s specific indicators, sum them up, and then find the corresponding value on the total score axis. They can then vertically locate the recurrence risk axis to quickly obtain the probability of postoperative hematoma recurrence for that patient. Through detailed assessment using nomograms, the clinical team can formulate targeted postoperative management strategies, such as extending drainage time, strengthening imaging monitoring, or discussing the feasibility of hyperbaric oxygen therapy based on the patient’s condition.

Compared with the prediction models in previous studies, the nomogram in this study has significant advantages. Traditional models often focus on a single treatment method, and the variables included are limited to clinical characteristics such as age and underlying diseases, without considering surgery-related factors and treatment interventions. This model not only integrates key indicators before, during, and after surgery, but also innovatively incorporates hyperbaric oxygen therapy, an emerging intervention, into the assessment system, more comprehensively reflecting the therapeutic characteristics of MMA combined with burr hole drainage.^[27]^ In addition, the reliability and discriminatory power of the model were confirmed by internal validation using Bootstrap and good calibration curves and high AUC values. In clinical practice, nomograms can effectively assist in preoperative risk stratification, helping doctors identify patients at high risk of recurrence and formulate prevention strategies in advance. In the postoperative management phase, their predictive results can provide data support for drainage strategy adjustments and the timing of hyperbaric oxygen therapy. At the same time, visual prediction tools also facilitate communication between doctors and patients. Doctors can use intuitive charts to explain the condition and prognosis to patients and their families, thereby improving patient compliance with treatment plans and truly achieving accurate, individualized management of CSDH.^[25]^

### 4.3. Advantages of the recurrence model after MMAE combined with burr hole drainage in patients with CSDH

Previous studies on recurrence after MMA combined with burr hole drainage in patients with CSDH have mostly focused on single-dimensional risk factors. For example, some studies emphasize that advanced age, anticoagulant use, and brain atrophy are key factors leading to recurrence, which is consistent with the pathological characteristics of chronic patients, such as decreased blood vessel wall elasticity, coagulation mechanism disorders, and insufficient intracranial compensatory space. Other studies have focused on factors related to surgical procedures, such as incomplete MMAE and insufficient drainage tube retention time, but a systematic multi-factor analysis framework has yet to be established.^[25]^ Compared with these studies, this study broke through the limitations of single factors and constructed a more comprehensive risk assessment system by incorporating multidimensional indicators such as postoperative drainage volume, hyperbaric oxygen therapy, GCS score at admission, and preoperative hematoma volume. Secondly, this study significantly improved the reliability of the results and the stability of the model through multi-factor logistic regression analysis combined with the internal validation method of bootstrap resampling. For example, the high degree of conformity between the calibration curve and the ideal curve, as well as the AUC values of both the training set and the validation set being close to 0.9, confirm the robust performance of the model in this study across different datasets. This rigorous validation process has been rarely addressed in previous studies.

In addition, previous prediction models have mostly remained at the theoretical level, lacking intuitive clinical translation tools. This study innovatively constructed a nomogram, transforming complex multi-factor analysis into a visual prediction tool, significantly improving the clinical practicality of the model. For example, in clinical decision-making, doctors can use nomograms to quickly calculate the risk of recurrence in patients, thereby adjusting postoperative drainage strategies and formulating personalized hyperbaric oxygen therapy plans. This advantage is difficult to achieve with traditional risk models. In addition, this study is the first to include hyperbaric oxygen therapy in the analysis of risk factors for recurrence, revealing the balance between this intervention’s role in promoting hematoma absorption and its potential pro-angiogenic effects. This provides a new perspective for optimizing clinical treatment plans and fills a gap in previous research on the evaluation of emerging treatment modalities.

### 4.4. Research limitations and future prospects

Although this study systematically analyzed the risk factors for recurrence after MMA combined with burr hole drainage and constructed a predictive model, there are still many limitations. First, as a retrospective study, the data came from single-center clinical records, so there was inevitably some selection bias. For example, differences in the management of patients’ underlying diseases and perioperative medication regimens may arise due to the preferences of the treatment team, making it difficult to fully extrapolate the study results to different medical environments. Secondly, the study lacks long-term follow-up data and only focuses on short-term recurrence after surgery, while the risk of CSDH recurrence may persist and the long-term prognosis is affected by many dynamic factors. The existing conclusions cannot fully reflect the long-term evolution of the disease. In addition, although the model incorporates risk factors covering clinical, imaging, and treatment intervention aspects, it may still overlook potential influencing factors such as patients’ genetic background and inflammatory factor levels, thereby limiting further improvements in the model’s predictive accuracy. This study did not conduct a comprehensive examination of the fundamental assumptions underlying multiple logistic regression analysis, including the linearity of the logit, the absence of multicollinearity, and the independence of observations. The failure to verify these assumptions may have impacted the reliability of the research findings. For instance, the lack of linearity testing could lead to biased estimates of variable relationships within the model, thereby failing to accurately reflect the true associations between variables. Similarly, neglecting multicollinearity may have exaggerated the effects of certain variables, resulting in less precise interpretations of the outcomes. Future research should prioritize testing these assumptions, employing more rigorous research designs and analytical methods – such as increasing sample sizes and conducting sensitivity analyses – to further enhance the accuracy and reliability of findings. To address these limitations, future research could be conducted in the following directions. First, there is an urgent need for multicenter, large-sample prospective studies to reduce selection bias and enhance the generalizability of research results through standardized data collection and uniform intervention protocols. For example, collaborating with neurosurgical centers in different regions to include a broader patient population and validate the stability of existing risk factors under different medical resources and treatment practices. Secondly, exploring the predictive value of novel biomarkers and imaging features for recurrence risk. In recent years, it has been confirmed that the levels of inflammatory factors (such as interleukin-6 and tumor necrosis factor-α) in plasma and dynamic perfusion parameters on MRI are associated with hematoma evolution. Incorporating these indicators into predictive models may reveal potential pathological mechanisms and improve predictive accuracy.

## 5. Conclusions

Through systematic multifactorial analysis, this study clarified that postoperative drainage volume, hyperbaric oxygen therapy, GCS score at admission, and preoperative hematoma volume are independent risk factors for recurrence after MMA combined with burr hole drainage. Research has confirmed that precise management of postoperative drainage volume is closely related to the balance of hematoma cavity pressure. Hyperbaric oxygen therapy, as an intervention method, plays a positive role in reducing the risk of recurrence by improving brain oxygen metabolism and regulating inflammatory responses. The neurological status reflected by the GCS score upon admission and the mass effect caused by the preoperative hematoma volume both indicate that individual differences significantly affect the prognosis. The predictive model constructed based on the above findings provides clinicians with an intuitive and practical risk assessment tool. This model can help neurosurgeons quickly identify patients at high risk of recurrence before and during surgery, thereby formulating personalized treatment plans, such as optimizing drainage strategies and determining the timing of hyperbaric oxygen therapy. This achievement not only deepens our understanding of the mechanism of recurrence after combined treatment for CSDH, but also provides new evidence-based grounds for improving patient prognosis and reducing waste of medical resources, which is of great clinical value in promoting the precise management of CSDH.

## Author contributions

**Writing – original draft:** Wen Cheng, Quanlong Yang, Xiaodong Yuan.

**Writing – review & editing:** Wen Cheng, Jiangbin Wu.

## Supplementary Material


